# 支气管、隆凸及肺血管成形术治疗中央型肺癌92例临床分析

**DOI:** 10.3779/j.issn.1009-3419.2010.11.11

**Published:** 2010-11-20

**Authors:** 丰 邵, 如松 杨, 栋生 许, 卫 邹, 国栋 马, 珲 曹, 宴青 潘

**Affiliations:** 210029 南京，南京市胸科医院胸外科 Department of Thoracic Surgery, Nanjing Chest Hospital, Nanjing 210029, China

**Keywords:** 肺肿瘤, 袖状切除, 隆凸成形, Lung neoplasms, Sleeve resection, Carinal resection

## Abstract

**背景与目的:**

外科手术是早、中期非小细胞肺癌的首选治疗方案。本文总结支气管袖状、隆凸切除及支气管肺动脉双袖状成形术等手术方式治疗92例中央型肺癌的临床经验。

**方法:**

对我院1996年1月-2010年5月间92例中央型肺癌患者施行以支气管袖状成形术为主的多种切除重建手术。其中右肺上叶支气管袖状切除术49例，左肺上叶袖状切除术14例，右肺中叶袖状切除术3例，左肺下叶袖状切除术4例，左肺支气管肺动脉双袖状成形肺叶切除术8例，右肺上叶切除合并器官隆凸切除重建3例，全肺切除合并气管隆凸切除重建术7例，主气管袖状切除4例。

**结果:**

无围手术期死亡病例，平均手术时间2 h 43 min，平均失血415 mL，术后肺不张7例（7/92），声音嘶哑4例（4/92），机械通气支持3例（3/92）。1年、3年、5年生存率分别为80.7%、59.6%、31.5%。

**结论:**

隆凸切除、支气管袖状成形术、支气管肺动脉双袖状成形术等术式既能最大限度地切除肿瘤，又能最大限度保护了肺功能，且隆凸切除气道重建术能进一步扩大手术适应症，提高了中央型肺癌的手术切除率。

## Abstract (en)

**Background and objective:**

Surgery is the best treatment for early and middle stage non-small cell lung cancer. The aim of this study is to summarize the experience of bronchial sleeve mordality lobectomy and carinal resection in the treatment of 92 patients with central lung cancer from January, 1996 to May, 2010.

**Methods:**

A total of 92 patients with central lung cancer underwent pulmonary resection. Carinal resection and reconstruction were performed in 14 patients, bronchial sleeve resection in 70 patients, and bronchial sleeve combined with pulmonary artery sleeve lobectomy in 8 patients.

**Results:**

There was no operative mortality. The average operation time was 2 hours and 43 minutes. Postoperative complications such as pulmonary atelectasis occurred in 6.94% (7/92) of total group, and hoarseness in 4.35% (4/92). The 1, 3 and 5 year survival rates were 80.7%, 59.6% and 31.5%.

**Conclusion:**

Bronchial sleeve lobectomy and double sleeve lobectomy are capable of excising pulmonary tumor as much as possible while remaining healthy lung tissues. Carinal resection and reconstruction is helpful to extend the surgical indication, and increase the chance of successful resection.

目前，外科手术是早、中期非小细胞肺癌的首选治疗方案，随着麻醉水平以及手术技术的不断提高，支气管、隆凸、血管袖状切除成形术成为病变在支气管开口附近，或已侵犯叶支气管开口、甚至主支气管及隆凸部位时的最佳术式，其目的是彻底切除病变的同时保留更多的肺组织。本院1996年1月-2010年5月，采用支气管袖状、隆凸切除成形、支气管肺动脉双袖状切除等多种术式治疗中央型肺癌92例，效果较为满意，现报道如下。

## 1 材料与方法

### 1.1 一般资料

本组92例，男75例，女17例；年龄28岁-77岁，平均56.9岁。临床症状有咳嗽（74/92）、痰中带血（64/92）等。胸部CT显示肺内肿块或支气管内肿块，其中伴阻塞性肺炎或肺不张53例。纤维支气管镜（fiberoptic bronchoscopy, BF）检查见气管、隆凸、支气管内新生物77例，支气管充血、狭窄、分嵴增宽15例。肿瘤位于左上叶19例，左下叶4例，右上叶49例，中间支气管3例，右中叶3例，右主支气管及隆凸8例，左主支气管及隆凸2例，气管下段4例。术后病理分型：鳞癌63例，腺癌15例，低分化癌8例，乳头状腺癌3例，粘液表皮样癌2例，腺样囊性癌1例。TNM分期IIb期7例，Ⅲa期51例，Ⅲb期34例。

### 1.2 手术方式

本组92例患者的手术方式可以归纳为9种：①右肺上叶支气管袖状切除，右主支气管和中间支气管端端吻合术49例；②左肺上叶支气管袖状切除，左主支气管和下叶支气管端端吻合术14例；③右肺中叶袖状切除术3例，切除中叶及部分中间支气管，行中间支气管与下叶支气管端端吻合；④左肺下叶袖状切除术4例，袖式切除下叶，左上叶支气管与左主支气管端端吻合；⑤右全肺切除合并隆凸切除5例，主气管与左主支气管端端吻合，隆凸重建；⑥右上肺切除合并隆凸切除3例，右中间干支气管与左主支气管端侧吻合，主气管与左主支气管端端吻合，隆凸重建；⑦左全肺切除合并隆凸切除2例，主气管与右主支气管端端吻合，隆凸重建；⑧主支气管袖状切除术4例，主要用于支气管狭窄的病例，切除主支气管狭窄段，行主支气管端端吻合，不切除肺组织；⑨双袖状切除术8例，行左上叶袖状切除术，左主支气管和下叶支气管端端吻合，同时行左肺动脉袖状切除。

在支气管、隆凸切除后，采用4-0 Prolene线连续缝合或1^#^丝线间断缝合气管支气管残端。由于两端吻合口直径存在差异，应用针距调整法54例，远端支气管凹或凸弧形切面法31例，近端支气管倒“V”形切除缝缩法5例，远端支气管斜切法2例。吻合针距大约2 mm，吻合完毕吻合口纵隔胸膜包埋缝合并喷涂生物蛋白胶。支气管加肺动脉双袖状切除时，肺动脉吻合采用5-0 Prolene线连续外翻缝合，吻合过程中用肝素水反复冲洗管腔，防止血栓形成。

## 2 结果

本组无手术死亡病例，手术时间1 h 30 min-4 h，平均手术时间2 h 43 min，平均失血415 mL。术后未发生支气管胸膜瘘；术后并发肺不张7例（7.61%），床边BF检查发现吻合口处大量的粘稠的分泌物阻塞，予以生理盐水冲洗吸除，并配合超声雾化和抗生素治疗后均治愈。3例患者术后机械通气，其中2例隆突成形术后予以机械通气支持治疗，1例72岁老年患者右全肺切除合并隆凸切除术后发生严重肺部感染、呼吸衰竭，经机械通气辅助呼吸6 d后治愈。4例喉返神经损伤致声音嘶哑，其中3例发生于右肺上叶袖状切除术，另1例发生于左主支气管袖状切除术。本组92例患者随访时间1个月-85个月，支气管吻合口无明显狭窄。术后生存时间为6个月-7年，1年、3年、5年生存率分别为80.7%、59.6%、31.5%。

## 3 讨论

目前，肺癌已经成为威胁人类健康的首位恶性肿瘤，而外科手术仍是早、中期非小细胞肺癌的首选办法，肺癌外科的公认原则是最大限度地切除肿瘤组织和最大限度的保留健康肺组织。外科治疗中央型肺癌有时比较棘手，由于邻近的肺门淋巴结和血管等易受侵，手术困难，切除率低；另一方面，累及主支气管及上叶开口的中央型肺癌按常规方法往往须做全肺切除，影响生存质量及疗效，而且浸润到气管下端侧壁、隆凸部时，常规的全肺切除也不能达到根治目的。关于支气管袖状切除成形或支气管袖状肺叶切除术，国内在20世纪70年代末至20世纪80年代初开始报道。现在该术式在肺癌外科的临床应用中已经比较成熟^[1]^。Jean等^[2]^研究发现，肺袖状切除术与全肺切除术相比，肿瘤完全切除率高，手术死亡率低，肿瘤复发率低，术后5年生存率高。肺袖状切除术患者预后较好的原因可能是最大限度地保留了肺功能，为后继的肿瘤综合治疗提供了足够的肺功能支持。本组92例支气管、隆凸成形手术体会是，熟练掌握支气管及段支气管的解剖、选择合理的手术方式、使重建的呼吸道接近正常的生理解剖对手术的成功非常重要。对于恶性肿瘤，切除线至少距肿瘤边缘0.5 cm以上。吻合材料可选择4-0 Prolene线全层连续缝合，或者1号丝线全层间断缝合，针距大约2 mm左右，线结打在支气管腔外。肺动脉受侵曾是袖状切除的禁忌症，现采用支气管加肺动脉双袖状切除可获得较好的疗效。Yildizeli等^[3]^报道218例袖式切除肺癌患者中28例行血管袖式切除，死亡率为1.6%，其5年生存率为37.1%。本组8例采用支气管肺动脉双袖状成形术，肺动脉吻合采用5-0 Prolene线连续外翻缝合，吻合过程中用肝素水反复冲洗管腔，防止血栓形成。肿瘤顺利切除，术后未出现明显并发症，但由于肿瘤已经侵犯血管，病程较晚，其中1例术后16个月出现纵隔和脑转移。由于呼吸道重建，支气管内膜纤毛排送系统的功能下降以及吻合口出血水肿等因素，支气管成形术后最常见的问题是排痰困难，因此呼吸道的管理显得非常重要。在吻合前应将呼吸道内的痰液和血液吸净。术后常规BF观察吻合口情况，对于痰多且难以咳出或疑有肺不张的患者，应及时行BF检查、吸痰，有助于保持呼吸道的通畅，并促使余肺膨胀。

气管隆凸切除气道重建术是治疗累及主支气管近端、隆凸嵴和气管下段侧壁中央型肺癌的手术方法。因该部位解剖结构特殊，手术难度大，并发症多，手术死亡率高达13%-29%，所以此术式的普及受到了影响^[4]^。因为右侧主支气管明显短于左侧主支气管，右侧中央型肺癌容易侵犯隆凸及气管下段，右侧开胸术野及隆凸显露优于左侧，右侧袖式隆凸全肺切除较左侧易于操作与解剖，左侧袖式全肺隆凸切除由于主动脉弓的阻挡影响而造成术野显露较差，解剖困难，本组中右侧袖式隆凸全肺切除术明显多于左侧（5/2）。右全肺切除隆凸重建术适用于右肺中央型肺癌向上侵犯隆凸及气管下段、右中间干支气管受侵的病例，常采用右后外侧切口，切除右全肺、隆凸及或气管下段部分，气管支气管的切缘距瘤组织一般应大于1 cm，主气管与左侧主支气管行端端缝合，吻合口喷涂生物蛋白胶，术后无吻合口相关并发症的发生。左侧袖式全肺隆凸切除由于主动脉弓的阻挡影响而造成术野显露较差，解剖困难。本组行左隆凸全肺切除2例，通过游离松解主动脉弓可以适当扩大视野，于主动脉弓上完成吻合操作，吻合尚满意。由于隆凸切除术后咳嗽反射减低甚至消失，同时伴随肺迷走神经切断导致支气管去神经支配张力降低，小气道萎陷，加之吻合口水肿以及切口疼痛等因素致使肺内分泌物排出不畅，术后辅助咳痰尤为重要，必要时应主动行BF吸痰排除肺内分泌物以保持呼吸道通畅，加强呼吸道管理和抗感染治疗。本组11例患者术后1 d-2 d均给予主动BF吸痰，效果良好。1例72岁患者右全肺切除合并隆凸切除术后由于咳嗽排痰不畅，发生肺部感染、呼吸衰竭，经机械通气辅助呼吸6 d后治愈。

肺癌患者术后采用放疗、化疗等综合治疗可明显提高患者远期生存率。肺癌侵犯隆凸已属中晚期，经过隆凸切除与重建术虽然病灶已得到临床完全性切除，术后仍需辅以多学科综合治疗。本组92例支气管、隆凸切除患者术后常规行4个-6个周期化疗并免疫支持治疗，效果尚可，5年生存率为31.5%。随着外科手术和麻醉技术水平的不断提高，肺癌多学科治疗的逐步完善，在严格掌握手术适应症的前提下，对中央型肺癌进行支气管、隆凸成形术和术后多学科治疗，可最大限度地切除肺癌组织和最大限度地保留患者的肺功能，提高患者的术后生存率和生活质量。
